# Optically induced effective mass renormalization: the case of graphite image potential states

**DOI:** 10.1038/srep35318

**Published:** 2016-10-14

**Authors:** M. Montagnese, S. Pagliara, G. Galimberti, S. Dal Conte, G. Ferrini, P. H. M. van Loosdrecht, F. Parmigiani

**Affiliations:** 1 Dipartimento di Matematica e Fisica, Università Cattolica del Sacro Cuore, Brescia I-25121, Italy; 2 i-LAMP (Interdisciplinary Laboratory for Advanced Materials Physics) Università Cattolica del Sacro Cuore, Brescia I-25121, Italy; 3 Dipartimento di Fisica, Università degli Studi di Pavia, Pavia I-27100, Italy; 4 II. Physikalishes Institut der Universität zu Köln, Köln D-50937, Germany; 5 Dipartimento di Fisica, Università degli Studi di Trieste, Trieste I-34127, Italy; 6 Sinctrotrone Trieste S.C.p.A, Basovizza I-34012, Italy

## Abstract

Many-body interactions with the underlying bulk electrons determine the properties of confined electronic states at the surface of a metal. Using momentum resolved nonlinear photoelectron spectroscopy we show that one can tailor these many-body interactions in graphite, leading to a strong renormalization of the dispersion and linewidth of the image potential state. These observations are interpreted in terms of a basic self-energy model, and may be considered as exemplary for optically induced many-body interactions.

Many-body interactions strongly influence the behavior of electrons in low-dimensional material systems, owing to the intrinsically enhanced Coulomb interactions in reduced dimensions. Examples of this are found in the emerging superconductivity in the two dimensional (2D) electron gas at the LaAlO_3_/SrTiO_3_ interface^1^, and the competition between stripe order and high-Tc superconductivity in layered cuprates^2^. A common characteristic of these interacting ground states is that their properties can be controlled by means of an applied electromagnetic field, a highly desirable feat in light of applications in microelectronics. In this framework, the ongoing quest to find predicted many-body phenomena in topological insulators^3^,^4^ has also focused on understanding the interactions between the topologically-protected 2D state wrapping the surface and the bulk electronic structure, which represents the main bottleneck limiting the charge and spin transport at the surface of these materials^5^. More in general, the properties of a surface or interfacial state, such as its effective mass and scattering lifetime, are determined by the many-body interactions with the underlying bulk electronic structure. In principle, these properties could be controlled by modifying the bulk structure through the application of an external static or pulsed electromagnetic field. These efforts, however, are only at the beginning.

An excellent candidate to study induced many-body interactions is the archetypical two-dimensional *Image potential state* (IPS) formed on the surface of many conducting materials^6^,^7^. The IPS arises from the attractive image potential an electron experiences when placed close to the surface of a conducting material with an electronic bulk bandgap near the vacuum energy^8^. The IPS, which is empty at equilibrium, has a free-electron-like character along the surface and it is quantized into a pseudo-Rydberg series in the perpendicular direction. The relative simplicity of these states makes the IPS an ideal realization of a quasi-2D electron gas. In principle, one should also be able to control the IPS properties by manipulating the bulk electronic states. This seems most easily achievable through bulk photoexcitation using intense ultrashort light pulses. However, for most of the metal substrates studied so far this approach turns out to be challenging in view of the very short lifetime of the photoexcited states. Typically the photoexcited bulk electrons relax to a quasi-equilibrium state near the Fermi level within a few tens of femtoseconds – a time comparable or shorter than the typical IPS lifetime. Moreover, the three dimensional character of the bulk electronic states and the high carrier densities at the Fermi level effectively screen interactions between bulk and surface electrons, which is not favorable to manipulate the properties of the IPS in this manner.

Here, by using momentum resolved nonlinear photoelectron spectroscopy, we show that these challenges can be overcome by using graphite as conducting substrate, and that in this exemplary case one can indeed significantly manipulate the properties of the IPS at the surface by a photoinduced many-body interaction between the graphite π-band electrons and the IPS. This is evidenced by a substantial effective mass renormalization and a concomitant change in the IPS linewidth, and can be quantitatively interpreted within the framework of a simple semi empirical self-energy model.

The quasistatic properties of IPS have been extensively studied by means of nonlinear photoelectron spectroscopy. The IPS lifetime is determined by its coupling to excitations in the bulk, and is in the tens of femtoseconds range^9^. On clean metal surfaces the IPS effective mass nearly always reduces to the bare electron mass; apparent deviations observed on certain oriented metal surfaces have been accounted for in terms of spurious momentum-dependent IPS ground state energy variations due to a strong bandgap anisotropy^10^,^11^. To date, the IPS properties have been successfully modified by directly tampering with the *surface* electrostatic potential, either through introducing a periodic lattice of highly electronegative adsorbates, such as C_60_^12^, or by transiently photoemitting a dense hot-electron gas near the surface^13^.

Due to its layered structure, the electronic structure of graphite can be described in terms of quasi-2D stacks of π-orbital networks with a marked semimetal character. In the near-UV range, saddle points in the π-bands determine twin van Hove singularities (vHs) in the density of states within a 4 eV range around Fermi energy [see e.g. the band structure in Fig. 1]. This, and the reduced screening arising from the two dimensional semimetal character of graphite, enhances many-body effects, causing deviations from ideal Fermi liquid behavior^14^,^15^ and inducing for instance a static^16^ and a photoinduced^17^ bandgap renormalization (BGR) in its optical response. The layered character of graphite also has a strong influence on the nature of the IPS^18^. The IPS in graphite can be viewed upon as originating from the twin surface states which are supported by a single layer of graphene. As multiple graphene sheets are stacked to form bulk graphite, the surface states interact with the 2D electrons in each sheet to hybridize into an interlayer band in the bulk and the IPS at the surface^19^. This peculiar layered structure is thought to play a role in several low energy many-body effects, such as the anomalous superconductivity of Alkali-intercalated graphite^20^. Exploiting such an intimate surface-bulk connection, IPS have been successfully employed to investigate the *static* interaction between epitaxial single- and few layer graphene and underlying metal substrates^21^,^22^,^23^,^24^. Nonlinear photoelectron spectroscopy employing intense ultrashort laser pulses in the visible and near UV range provides an ideal tool to reveal the *dynamic* interactions between bulk graphite states and the IPS on graphite. Using this method one can induce high excitation densities in the bulk band states in a controllable way, while at the same time obtain a detailed picture of the momentum-energy dispersion of the electronic IPS states^25^.

## Results

**Figure 1:**
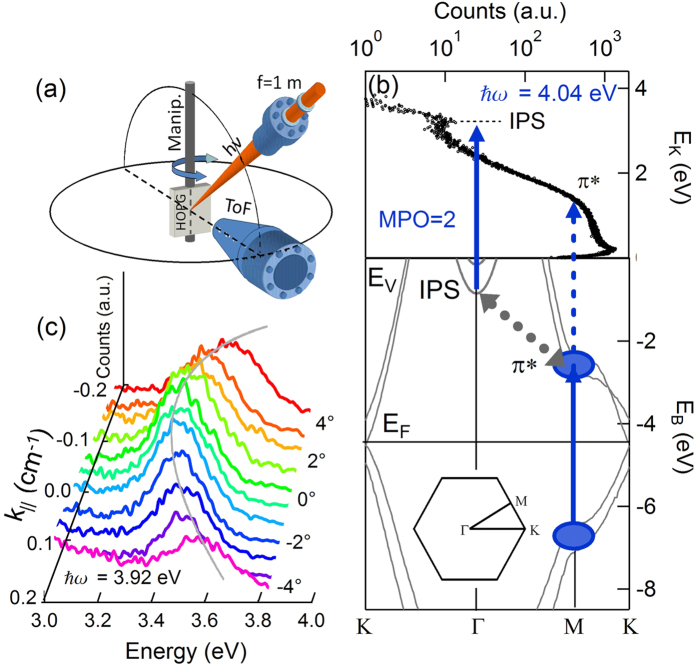
(**a**) Detail of the experimental setup (altitude geometry). The laser beam lying on the altitude plane is focused on the sample by an external f = 1 m lens. The azimuthal plane is also indicated by a circle. The ToF axis is indicated by a dashed black line. (**b**) Normal emission photoelectron spectrum. The IPS and the π-band shoulder (πS) are highlighted along with the excitation-photoemission pathways. The bulk electronic structure and the surface BZ are shown. (**c**) ationTypical IPS dispersion taken at 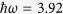 eV. The emission angle is indicated on the graph right side. The gray parabolic line is a guide for the eye.

The upper part of Fig. 1(b) shows a normal emission spectrum taken with 4.04 eV photons. The peak located around 3 eV originates from the n = 1 IPS, while the hump-shaped feature (π*) located in the 1–2 eV range is assigned to the linear photoemission from the anti-bonding π* band, transiently populated via a π→π* transition in the bulk [bottom of Fig. 1(b)]. This assignment is confirmed by the polarization selection rules and by the measurement of the multiphoton order (MPO) for both features, i.e. the total number of photons employed to populate and photoemit the feature. The details of the population mechanism for the IPS and the π* shoulder have been discussed in a recent paper^26^, where it is shown that in graphite the IPS is populated through the same π* electron population that produces the π* shoulder, via an unusually efficient indirect transition. The efficiency of the population process is maximized using 4.0 eV photons. This corresponds to the vHs peak in the imaginary part of the dielectric function of graphite, redshifted by a photoinduced BGR of 0.4 eV with respect to the static resonance^17^. Figure 1(c) shows a typical IPS dispersion taken at 3.92 eV photon energy. For each emission angle, the IPS raw spectra have been fitted with a Voigt curve and an exponential background to extract IPS intensities, kinetic energies, and linewidths.

**Figure 2:**
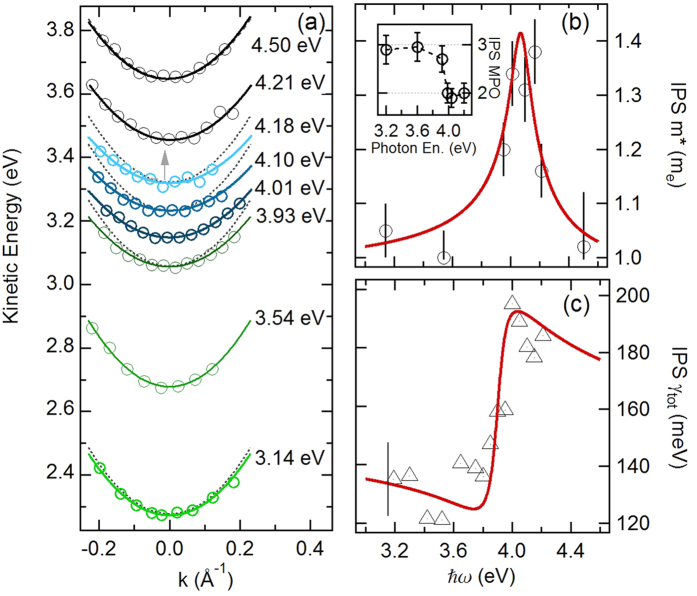
(**a**) IPS dispersions at various photon energies (open circles) with the relative parabolic fits. Dashed parabolas represent the free electron dispersion for comparison. (**b**) The effective mass data extracted from the dispersions are shown versus photon energy. Inset: the IPS multiphoton order (MPO) at different photon energies. The solid red line is a fit of Equation 5. (**c**) IPS linewidth at normal emission versus photon energy (open triangles). The solid red line is the fit to Equation 6.

The extracted kinetic energy versus parallel momentum are shown in Fig. 2(a) where several angle-resolved IPS dispersions, collected at various photon energies in the 3.14–4.50 eV range, are reported. For photon energies far from resonance, the IPS is mostly populated via a two-photon process from the π band edge and subsequently directly photoemitted [resulting in a MPO of three, see inset of Fig. 2(b)]; the IPS dispersion is consistent with a free electron mass (dashed parabola). At photon energies around 4.0 eV the IPS MPO reduces from three to two, indicating that the IPS can now be directly populated by the high density of photoexcited carriers at π^*^ band van Hove singularity [see Fig. 1(b)], via an indirect transition, possibly enhanced by the high density of excited carriers at the M point^26^. Notably, in the same energy interval, the IPS dispersion significantly flattens indicating an appreciable increase of the effective mass.

The extracted effective masses are shown in Fig. 2(b) as a function of the photon energy, while the IPS linewidths at normal emission are shown in Fig. 2(c). The effective mass reaches a maximum of 1.4 m_e_ around ħω = 4.0 eV; this is accompanied by a 45% increase of the IPS linewidth, i.e. from the previously reported value of 130 meV^18^ to about 190 meV. It is worth noting that on certain metal surfaces, a photoinduced hot electron gas in the unoccupied d-band manifold near the vacuum energy can scatter with the IPS band, violating its photoemission selection rules and inducing a small (about 9%) effective mass renormalization^27^.

## Discussion

This effect is due to an energetic overlap of the IPS with the long tail of the quasi-thermal energy distribution of the hot electron gas, which itself appears directly in the photoemission spectrum. In the present case, the mechanism responsible for causing the IPS modifications in graphite appears to be of another nature, since no dipole selection rule is violated and the optical π→π^*^ excitations are located well below the vacuum energy and are separated from the IPS in both energy and momentum (see Fig. 1b). This is confirmed by the fact that, beyond the relatively narrow π* shoulder at lower energies, no hot electron gas tail is visible in the photoemission spectra in proximity of the IPS feature. At the same time the effects on the IPS are much stronger than those due to interaction with a hot electron gas, with a 40 percent renormalization in the effective mass and a 45 percent linewidth increase.

This combined mass and linewidth renormalization points to a strong photoinduced many-body interaction, caused by the breakdown of Coulomb screening due to the high density of the photoinduced electron-hole population in the π-π* bands. Based on these assumptions, the IPS properties can be described by a simple self-energy model, taking into account its interaction with the photoexcitations in the bulk. In the shell approximation we can write


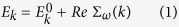



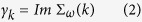


Here the IPS dispersion *E*_k_ and intrinsic linewidth γ_k_ are described in terms of the IPS self-energy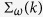 and the free electron dispersion 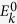
28. In order to determine the functional form of the self-energy we consider that: i) since the IPS mainly decasys to the underlying bulk, its dispersion and lifetime are determined by the details of the bulk electronic structure between vacuum and Fermi energy; ii) the *changes* in the IPS dispersion will be determined by the corresponding photoinduced *changes* to the bulk electronic structure; iii) such modifications, as discussed above, mainly consist of the π-π* bandgap renormalzation at the saddle point, *which scales as the square of the excitation density N*. We will therefore assume that, to the lowest approximation, the functional form for 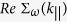 will be proportional to *N*^2^, and therefore fix it to be of the form:


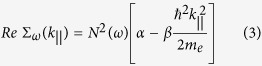


where


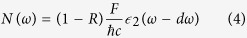


is the photoexcitation density in the π bands; we have used the theoretical expression for 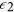 by Pedersen^29^. Here *dω* ∼ 0.4 eV is the dynamic BGR redshift fixed from optical measurements^26^. A reflectivity R = 0.33 has been used here^30^. It is worth noting that from Equation (4) we get that for the absorbed fluence of 150 μJ cm^−3^ at the resonance peak induces a carrier density of approximately 10^20^ cm^−3^, i.e. two order of magnitude higher than the equilibrium carrier density at room temperature^31^, indeed hinting to the non-perturbative regime for the bulk excitation. Here, *α* and *β* are the two energy-independent parameters of the model: *αN*^2^(*ω*) represents the *rigid energy shift* of the dispersion, whereas 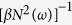 represents the *fractional effective mass increase* at the each photon energy, both being proportional to the many-body residual interactions in the bulk. From Equation 3 is possible to obtain the effective mass dependence on the photon energy:


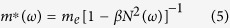


The contribution to the IPS linewidth at normal emission can be obtained from the imaginary part of the self-energy 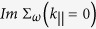 using Equation 3 and the Kramers-Kronig relations. The total linewidth, taking the experimental resolution (Γ_0_ = 70 meV) into account, is then given by


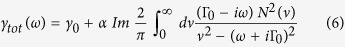


where *γ*_0_ = 130 meV is the equilibrium IPS bandwidth. A fit of Equation 5 to the data in Fig. 2b using the coupling constant *β* and the BGR shift *dω* as fitting parameters shows an excellent agreement with the experiment. The resulting fit for the BGR shift gives *dω* = 0.40 ± 0.05 eV, i.e. a value consistent with the photoinduced BGR shift measured from optical experiments^17^. Figure 2(c) shows a fit of Equation 6 to the linewidth data, with the BGR shift *dω fixed* from the fit of the effective mass, and *α* the only remaining fitting parameter. Our data and the excellent agreement with a simple self-energy model strongly points to photoinduced many-body interactions between the *π*−π* excitons and the IPS in graphite as the cause of the observed changes in the IPS effective mass and linewidth.

The choice of the density square power law *N*^2^ in the pre-factor in Equation 3 is fully consistent with our data. In principle, the *N*^2^ dependence could further be confirmed using experiments in which the laser fluence varies by one or two orders of magnitude. In practice, the fluence range of such experiments is limited by space charge broadening of the photoemission features at high fluencies and by low photoelectron statistics at low fluencies. The present model, however, has been validated over a narrower fluence interval by coupling the laser beam to the sample through the azimuthal geometry. In this way, as the manipulator angle *θ*_mp_ is varied in the ±25° range, it is possible to induce an incident fluence variation 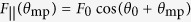. where *θ*_*0*_ = 30° is the angle between the excitation laser beam and the ToF axis. This approach reduces the fluence at one end of the dispersion curve to 30 percent of its value at the other side. This induces a measurable, asymmetric distortion in the IPS dispersion curves that can be fully reproduced with our model by inserting 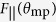 in Equations 3, 4, 5, thus confirming the model and in particular the nonlinear dependence of the photoinduced IPS on the photoexcited excitation density (see Supplementary information Fig. S2)

The strong interaction between the IPS dispersion and the bulk photoexcitation reported in this Letter finds its origin in the strong IPS-bulk coupling arising from the interlayer character of both the *π* band excitations and the IPS in graphite. On the other hand, photoinduced excitations at the π band saddle point have already proven to strongly deviate from the Fermi-liquid behavior, confirming the peculiar character of the M point of the BZ in graphite and its importance in determining the electrodynamics of graphite as a whole. In addition, surface states have been proposed to take a relevant part in the charge, spin and energy transport in single and multilayer graphene^32^; they also play an important role in determining the collective conduction properties of ensembles of graphene-derived compounds such as fullerenes and carbon nanotubes^33^. More recently, graphene sheets have been employed in novel devices exploiting two-dimensional electrons to manipulate THz light^34^; controlling the properties of IPS in graphene by means of light could enable to increase the capabilities of such devices. In conclusion, by using momentum resolved nonlinear photoelectron spectroscopy and laser pulses in the near UV energy range we could induce a strong many-body interaction between the bulk excitations and the image potential state in graphite. The resulting photon energy dependence of IPS photoemission effective mass and linewidth has been attributed to many-body interactions between π band excitons and the IPS when the exciting photon energy approaches the dynamically renormalized π band saddle point. These findings demonstrate the use of the IPS as a sensitive, non-perturbing probe for the many-body dynamics in materials, brings a clear evidence of a high IPS-bulk coupling in graphite, possibly due to its layered character. Finally they could open the way to manipulate the transport properties of surface states through the controlled induction of optical excitations in the bulk.

## Methods

The nonlinear photoemission experiment reported here is based on an amplified Ti:Sapphire laser (1 KHz repetition rate) and an optical parametric amplifier^26^. *P*-polarized, 100-fs, near-UV pulses in the 3.1–4.5 eV energy range are focused on a highly oriented pyrolytic graphite sample (HOPG, from MaTecK GmbH, Jülich, Germany. Mosaic spread better than 0.5°) sample kept in ultra-high vacuum (pressure lower than 2 × 10^−10^ mbar). Photoelectrons are detected by a custom-built time of flight (ToF) electron energy analyzer with an angular acceptance of ±0.85° and an overall energy resolution of 35 meV at an electron kinetic energy E_K_ = 2.0 eV^35^. Absorbed laser fluences are kept in the 150 μJ cm^−3^ range to avoid space charge distortions in the photoelectron spectra. The photoemission setup and the laser coupling geometry into the vacuum chamber are shown in Fig. 1(a). The laser beam lies in the altitude plane defined by the manipulator and the ToF axis (‘altitude geometry’) instead of in the azimuthal plane (‘azimuthal geometry’) in order to minimize fluence variations caused by geometrical effects and to maintain a constant excitation density as the sample is rotated (see Supplementary information Fig. S1). The HOPG samples are cleaved *ex-situ* and annealed at 450 °C in UHV until a good LEED pattern is observed. The photoelectron spectra have been collected as a function of the electron momentum parallel to the surface, *k*_||_. All experiments have been performed at room temperature.

## Additional Information

**How to cite this article**: Montagnese, M. *et al*. Optically induced effective mass renormalization: the case of graphite image potential states. *Sci. Rep*. **6**, 35318; doi: 10.1038/srep35318 (2016).

## Supplementary Material

Supplementary Information
