# Supplementary material for: Optically induced effective mass renormalization: the case of graphite image potential states
Source: Sci Rep. 2016 Oct 14;6:35318. doi: 10.1038/srep35318 (PMC5064354; doi:10.1038/srep35318)
Supplement: Supplementary Information [file srep35318-s1.pdf]

Supplementary Information for

Optically induced effective mass renormalization: the case of graphite image potential states

M. Montagnese[1*], S. Pagliara[1,2], G. Galimberti[1,2], S. dal Conte[3†], G. Ferrini[1,2], P.H.M. van Loosdrecht[5] and F. Parmigiani[4,5,6]

1-Dipartimento di Matematica e Fisica, Università Cattolica del Sacro Cuore, Brescia I-25121, Italy

2- i-LAMP (Interdisciplinary Laboratory for Advanced Materials Physics) Università Cattolica del Sacro Cuore, Brescia I-25121, Italy.

3-Dipartimento di Fisica, Università degli Studi di Pavia, Pavia I-27100, Italy

4-Dipartimento di Fisica, Università degli Studi di Trieste, Trieste I-34127, Italy

5- II. Physikalishes Institut der Universität zu Köln, Köln D-50937, Germany

6-Sinctrotrone Trieste S.C.p.A. Basovizza I-34012, Italy

The model employed in this Letter can also explain the even more surprising IPS dispersion behavior encountered when the non-symmetric experimental geometries (´A´ and ´B´ in Figure S.1) are employed to collect angle-resolved spectra around the 4 eV resonance.

Figure S.2(b) reports four dispersions, collected with various photon energies and different experimental geometries displayed in Figure2(a). In particular, the first dispersion shown $\hbar\omega = 4.04$ (eV, A geometry) and the second dispersion ($\hbar\omega = 4.26$ eV, B geometry) shows a remarkable asymmetry with respect to the $k_{||} = 0$ vertical axis, with one (´flat´) side of the curve dispersing noticeably less than the other (´steep´) side. In particular, the *flat* (*steep*) side of the dispersion is obtained when the sample is rotated *towards* (*away from*) the normal incidence condition. In fact, when the experimental geometry is mirrored by going from A to B, the flat and steep sides also flip. Even more strikingly, the symmetric character of the IPS dispersion is restored by *either* lowering the photon energy (see third dispersion, $\hbar\omega = 3.4$ eV photon energy, A geometry) *or* by employing the symmetric C geometry (see Figure S.1) with photon energy kept in the 4 eV range (fourth dispersion, $\hbar\omega = 3.93$ eV).

These dispersion anomalies can be explained in the framework of the model presented in this Letter, by carefully considering the variations in the absorbed fluence of the polarization component perpendicular to the sample surface $F_{||}$when the sample is rotated during a dispersion measurement. The angle dependence of $F_{||}$ induces changes in the photoinduced excitation density $N(\omega)$ in the π bands. This in turn induces a dependence on the manipulator angle of the IPS effective mass, which is encoded in the dispersion measurement as apparent parallel momentum dependence. Such an effect gets stronger as

Present addresses:

[*] II. Physikalishes Institut der Universität zu Köln, Zülpicher Str. 77, Köln D-50937, Germany

[†] IFN-CNR, Dipartimento di Fisica, Politecnico di Milano, Piazza L. da Vinci 32, Milano I-20133 Italy.

Electronic address: m.montagnese@ph2.uni-koeln.de

the angle nears normal incidence, where the fluence is maximal, increasing the effective mass and thus yielding a flatter dispersion.

This analysis can be made more quantitative by calculating the angle dependent fluence. The rotation of the manipulator determines two separate effects that depend on the angle of incidence (AOI) θ_i: i) a variation of the *area* of the sample illuminated by the laser beam

$$A(\theta_i) \sim cos(\theta_i); \quad \text{(S.1)}$$

and ii) a variation on the intensity of the polarization component of the laser parallel to the surface responsible for the π - π* transition:

$$I_{||}(\theta_\mathrm{i}) \sim \cos^2(\theta_\mathrm{i}). \quad \text{(S.2)}$$

In this way, the angle dependence of $F_{||}$ turns out to be

$$F_{||}(\theta_\mathrm{i}) = \frac{I_{||}(\theta_\mathrm{i})}{A(\theta_\mathrm{i})} \sim \cos(\theta_\mathrm{i}) \quad \text{(S.3)}$$

This is a general result valid for each of the experimental geometries. What differs between the two symmetries is the relationship between the AOI and the manipulator angle θ_{mp}. In the asymmetric A and B experimental geometries we have

$$\theta_\mathrm{i} = \theta_0 + \theta_\mathrm{mp}, \quad \text{(S.4)}$$

Where $\theta_0 = +30°$ ($\theta_0 = -30°$) is the azimuthal angle determining the A (B) geometry; for the symmetric C geometry we have

$$\cos\theta_\mathrm{i} = \cos\phi_0 \cos\theta_\mathrm{mp}, \quad \text{(S.5)}$$

where $\phi_0 = 45°$ is the elevation angle of the C geometry (see Figure S.1). The dependence of the fluence on the parallel momentum are plotted in Figure S.2(c) as empty circles. For the asymmetric geometries the fluence dependence is essentially linear with $k_{||}$ and its relative variation during a θ_{mp} sweep is substantial (i.e. more than 50%). For the symmetric geometry, however the dependence is quadratic in $k_{||}$, and showing little (about 2%) variation along the manipulator sweep.
From these considerations we obtain the momentum-dependent excitation density (*cf.* Equation 4 in the Letter):

$$N(\omega, k_{||}) = \frac{F_0}{\hbar c}\cos(\theta_i)\epsilon_2(\omega - d\omega) \quad \text{(S.6)}$$

were θ_i depends on the parallel momentum $k_{||}$ via the manipulator angle θ_mp. F_0 is the fluence at normal incidence. Equation S.6 is then used in Equation 1, via Equation 3 (see Letter), which is subsequently fitted to the experimental data to extract the parameter β. Results of the fitting procedure are reported in Figure S.2(b) as gray curves. The agreement between the IPS dispersion data and the fitting is very good for all the four dispersions.

Using the fitted parameter β, the momentum-dependent effective mass can now be obtained, as:

$$m^* = \frac{m_e}{1-\beta N^2(\omega,k_{||})}. \tag{S.7}$$

The resulting effective masses are plotted as full circles in Figure S.2(c). In particular, the fluence and effective mass curves show that the asymmetric character of the dispersion obtained with A and B geometries with photon energy in the 4 eV range can be explained by taking into account the variations of the excited carrier density due to the angle-dependent fluence variations induced by the particular beam-steering geometry. The extracted effective mass data shows a higher m^* for increasing fluence. This causes the IPS dispersion to locally flatten.

The model also fits well the third dispersion, taken with $\hbar\omega = 3.14$ eV photons and with the A geometry. In this case, even if the fluence, as expected, varies substantially within the parallel momentum interval considered, the dispersion is fairly symmetric; the extracted effective mass shows only a limited (about 2%) variation. This suggests that the IPS-π^* interaction strength is quenched when out of resonance (i.e. far from $\hbar\omega = 4.0$ eV), thus leaving substantially unaltered the IPS properties, as already confirmed by normal emission measurements.

The analysis of the fitting results of the fourth, $\hbar\omega = 3.93$ eV dispersion is even more illuminating in this respect, since it shows that with the symmetric geometry the incident fluence momentum-dependent variations have a symmetric character with respect to the $k_{||}$ axis and are less than 5%; the extracted effective mass shows a comparable behavior, being nearly constant at about 1.3 m_e. This confirms that the effective mass data collected with the C geometry are unaffected by spurious geometrical contributions due to the asymmetric experimental geometry employed.

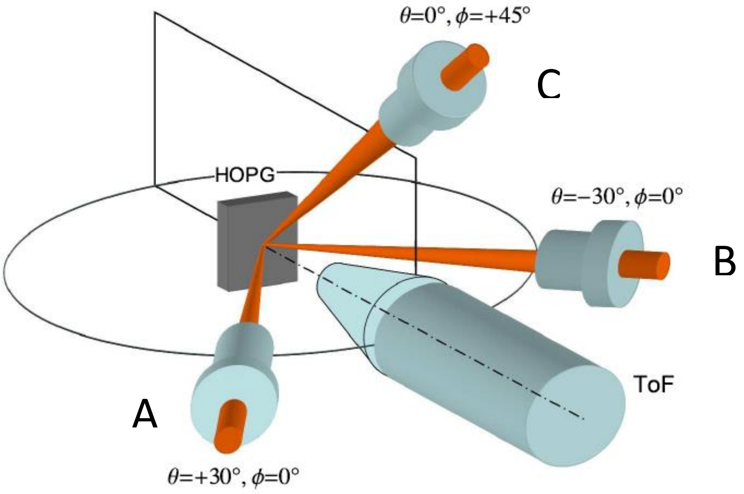

Figure S.1: (color online) experimental geometries. The azimuthal (A and B) and altitude (C) geometries are shown.

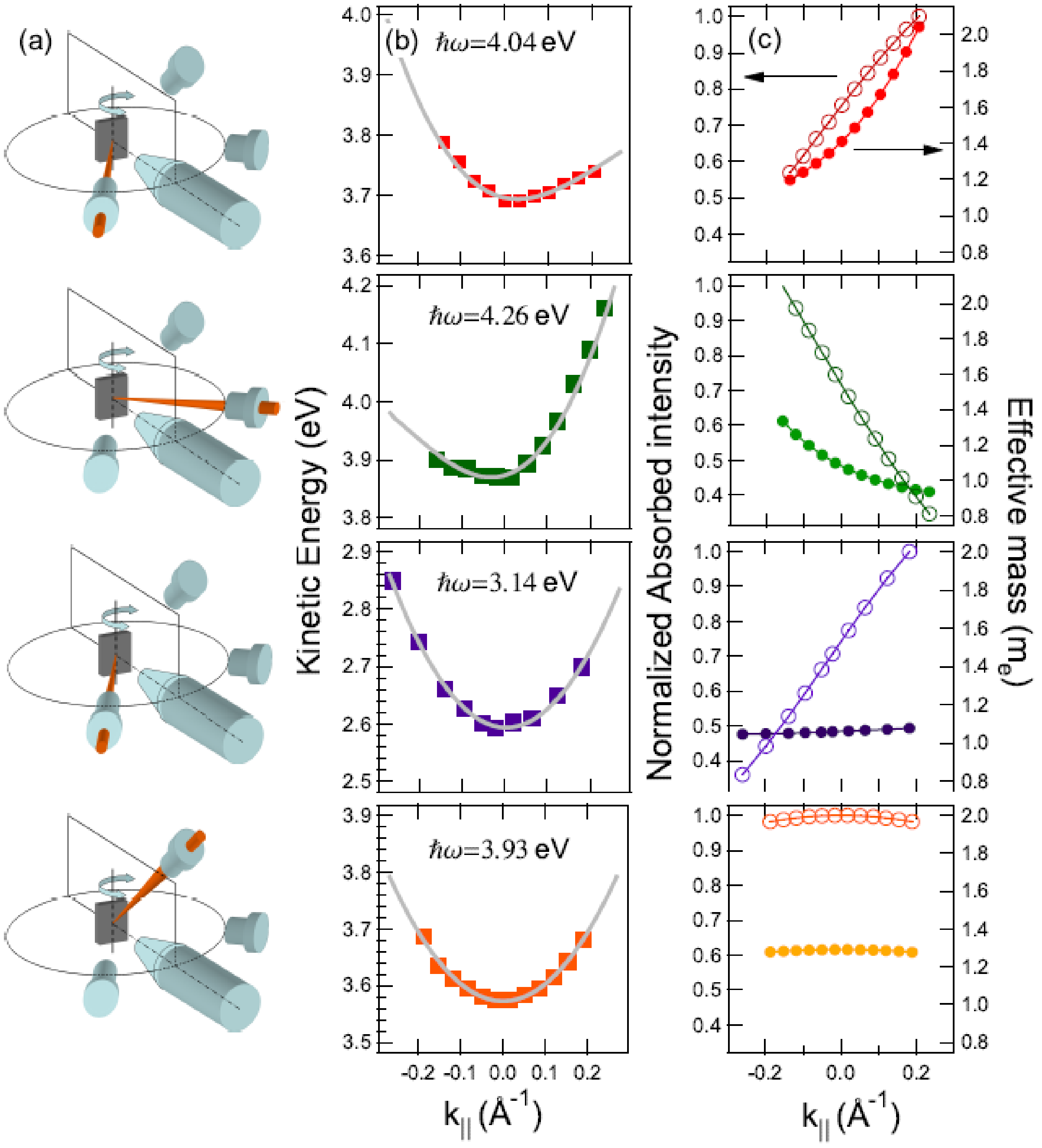

Figure S.2 : (color online) Four IPS dispersion taken with different geometries and different photon energies. Column (a): sketch of the experimental geometry employed; (b): measured dispersion (full square) and dispersion fit (gray line); (c) empty circles: normalized momentum-dependent fluence; full circles: momentum-dependent effective mass obtained from Equation (S.7) and using the value for the β parameter from the dispersion fit.

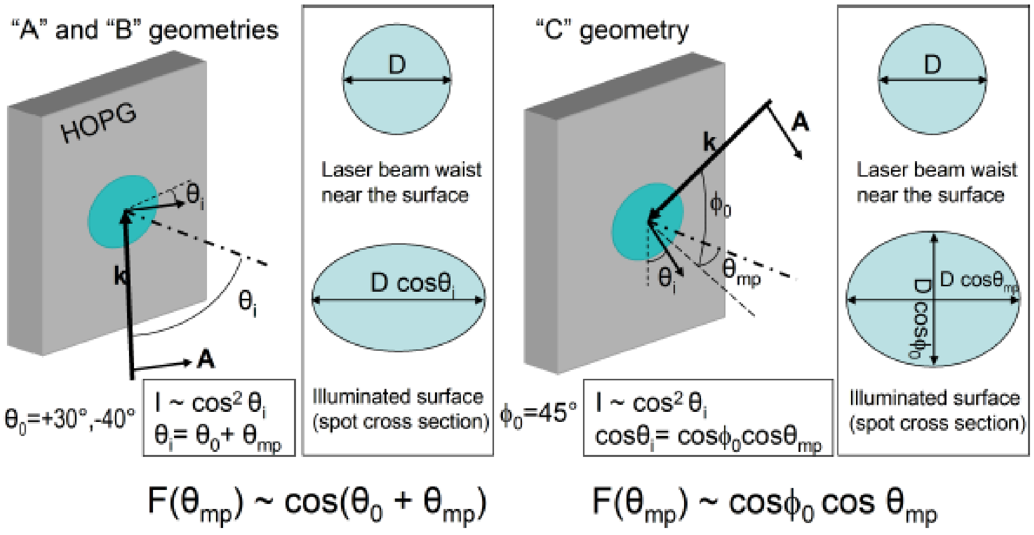

Figure S.3: (color online) The angle- dependence of the incidence fluence F in the different experimental geometries. Here I is the intensity of the polarization component of the laser vector potential A perpendicular to the surface considering a P- polarized laser beam of wave vector $\underline{\boldsymbol{k}}$; θ_i is the AOI, $\boldsymbol{\theta_0}$ ($\boldsymbol{\phi_0}$) is the geometry azimuthal (elevation) angle; $\boldsymbol{\theta_{mp}}$ is the manipulator angle.
